# Response – Building on existing knowledge and redefining rather than abandoning the well‐established ‘clinical high risk for psychosis’ prevention paradigm

**DOI:** 10.1111/camh.12777

**Published:** 2025-03-24

**Authors:** Gonzalo Salazar de Pablo, Claudia Aymerich, Grace Frearson, Javier de Otazu Olivares, Ana Catalan

**Affiliations:** ^1^ Department of Child and Adolescent Psychiatry, Institute of Psychiatry, Psychology & Neuroscience King's College London London UK; ^2^ Child and Adolescent Mental Health Services (CAMHS) South London and Maudsley NHS Foundation Trust London UK; ^3^ Department of Child and Adolescent Psychiatry, Hospital General Universitario Gregorio Marañón School of Medicine, Institute of Psychiatry and Mental Health Universidad Complutense, Instituto de Investigación Sanitaria Gregorio Marañón (IiSGM), CIBERSAM Madrid Spain; ^4^ Psychiatry Department, Biocruces Bizkaia Health Research Institute, OSI Bilbao‐Basurto, Facultad de Medicina y Odontología, University of the Basque Country UPV/EHU Centro de Investigación en Red de Salud Mental (CIBERSAM), Instituto de Salud Carlos III Barakaldo Bizkaia Spain; ^5^ School of Medicine Universidad Nacional Pedro Henríquez Ureña Santo Domingo Dominican Republic; ^6^ Department of Psychosis Studies, Institute of Psychiatry, Psychology & Neuroscience King's College London London UK

**Keywords:** Prevention, clinical high risk for psychosis, evidence

## Abstract

We appreciate the commentary by Tiffin and Kelleher on our systematic review and meta‐analysis. The CHR‐P paradigm remains one of the most established preventive approaches in mental health. While concerns have been raised regarding the clinical utility of the CHR‐P paradigm, its implementation in specialized services worldwide supports its relevance. These services provide evidence‐based interventions, reducing unnecessary antipsychotic use and guiding treatment strategies. Tiffin and Kelleher's critique largely focuses on transition rates and age cutoffs. While we argue that transition rates in adolescents at CHR‐P are significant, other outcomes need to be considered. Among others, negative symptoms are clinically significant in adolescents at CHR‐P, impairing functioning and long‐term outcomes. We think we should refine and improve the CHR‐P paradigm rather than simply abandoning it. With advancements in precision medicine, we can improve risk stratification and tailor interventions to better serve individuals at risk. We can also expand the paradigm, so it supports other help‐seeking adolescents at risk requiring transdiagnostic, developmentally sensitive interventions to prevent psychosis.

We are thankful to Tiffin and Kelleher for taking the time to read our work and for their commentary on our systematic review and meta‐analysis (Frearson, de Otazu Olivares, Catalan, Aymerich, & Salazar de Pablo, 2025). We also appreciate their acknowledgment of the strengths and weaknesses of the clinical high risk for psychosis (CHR‐P) paradigm, as well as their insights on some of the latter.

The CHR‐P paradigm represents probably the most established preventive approach in mental health (Salazar de Pablo & Arango, 2023). Three main groups within CHR‐P have been defined among young people (including adolescents and young adults): (1) Attenuated psychotic symptoms (APS)—individuals with recent subthreshold, attenuated psychotic symptoms; (2) brief limited intermittent psychotic symptoms (BLIPS)—individuals with short but frank psychotic symptoms; and (3) genetic risk and deterioration (GRD) syndrome—individuals with a first‐degree relative with a psychotic disorder or a personal history of schizotypal personality disorder, in addition to a decreased functioning during the previous year (Salazar de Pablo & Arango, 2023).

Tiffin and Kelleher report that the ‘CHR‐P’ paradigm is "obscure" and lacks clinical utility and relevance. The prevalence of the CHR‐P state has been meta‐analytically estimated as 1.7%, while in help‐seeking clinical samples including outpatient and inpatient clinics, the prevalence increases to 19.2% (Salazar de Pablo, Woods, Drymonitou, de Diego, & Fusar‐Poli, 2021). Since several CHR‐P services have been implemented worldwide to try to meet the needs of this non‐negligible number of young people at CHR‐P (Salazar de Pablo, Estradé, Cutroni, Andlauer, & Fusar‐Poli, 2021), the establishment of CHR‐P services is, per se, an indication of clinical utility or at least relevance. In these services, individuals fulfilling one or more of the aforementioned criteria have in the last decades received evidence‐based interventions such as psychoeducation, cognitive behavioral therapy and different medications (Salazar de Pablo, Estradé, et al., 2021). Furthermore, through clinical research in CHR‐P services, a decrease in the use of antipsychotics and replacement by other better tolerated medications with similar efficacy (e.g. antidepressants) has been achieved and recommendations included in clinical guidelines such as NICE guidelines (Fusar‐Poli & Salazar de Pablo, 2022a). As highlighted, adolescents at CHR‐P are typically help‐seeking individuals, experiencing distress associated with their experiences. We would thus consider providing them with need‐based interventions as clinically useful and relevant.

The main critique from the authors is related to the paradigm and particularly to transition to psychosis rates. We have already expressed our views in another commentary (Fusar‐Poli & Salazar de Pablo, 2022b) including our estimations that adolescents identified as at CHR‐P have a 23% risk of developing psychosis within 2–3 years, which is almost 100 times higher than the 0.3%–0.5% lifetime risk in the general population (~0.015%–0.02% per year). Briefly, while authors criticize including studies with any participant over 18 years, the reality is that the transition rates reported in their commentary are remarkably lower than those reported by any of the other previously published systematic reviews and meta‐analyses in the field, not only ours (see the full list in Fusar‐Poli & Salazar de Pablo, 2022b). This is because within their 436 individuals in total, individual analyses rely on only four studies per time point. In small powered meta‐analysis, findings can be unstable, and one study can change the results. Including a study with individuals with schizotypal personality disorder without the required functional deterioration to meet the criteria for CHR‐P could have caused this effect (Fusar‐Poli & Salazar de Pablo, 2022b). We believe that a better meta‐analytic approach to overcome the limited statistical power while at the same time testing the prognostic validity of the age cutoff at 18 years was to conduct sensitivity analyses and meta‐regressions to prove there are no differences in results or validity. That way, in several meta‐analyses, we have demonstrated that age is not a significant modulator of clinical outcomes, including transition to psychosis but also attenuated positive symptoms, negative psychotic symptoms, depressive symptoms, or remission (Fusar‐Poli & Salazar de Pablo, 2022b).

Although authors may argue that CHR‐P lacks enough specificity, this challenge is not unique to this field. For example, prediabetes is widely accepted as a clinical stage warranting intervention, despite the fact that not all individuals with prediabetes will develop diabetes. Similarly, CHR‐P should not be dismissed simply because not every young person will transition to psychosis. Rather, it should be refined. For this, the paradigm or the provision of preventive clinical care to adolescents at risk could be expanded in line with Tiffin and Kelleher's points. Another approach would be to use advances in genetics, neuroimaging, and machine learning to better stratify risk and personalize care.

The authors criticize opaque recruitment and characterization strategies in the CHR‐P field. We have previously detailed what these strategies are (Salazar de Pablo, Estradé, et al., 2021). We would argue that these have been better reported and are more transparent than in most mental health fields, where self‐reported symptom checklists are provided or referral pathways are non‐standardized and depend on clinical judgment or, worse, on waiting times. CHR‐P status and the presence of these three groups are assessed using validated interviews such as the ‘Comprehensive Assessment of At‐Risk Mental States’ (CAARMS) or the ‘Structured Interview for Psychosis‐risk Syndromes’ (SIPS) where the severity, the duration, and the pattern of presenting unusual experiences and psychotic symptoms are rigorously assessed. Often only individuals fulfilling CHR‐P criteria according to one of these instruments, with scores within certain limits, are accepted into these services. This leads to approximately one out of three referrals received by CHR‐P services being accepted and the rest being considered below or above the threshold. For adolescents above the threshold, clinical services for first‐episode psychosis providing early intervention strategies may be offered, while those below the threshold are often discharged back to their GP or Child and Adolescent Mental Health Services (CAMHS) provisions (Salazar de Pablo & Arango, 2023). We agree that this approach leaves the needs of a significant number of young people unmet, but why remove the support available when we can think about ways to help those young people not meeting the criteria for CHR‐P? As per recruitment strategies, briefly, in CHR‐P services, within active strategies, workshops for referral sources were the most frequent strategies (97.6%), often targeting healthcare professionals (85.4%), educational professionals (48.8%), or community organizations (34.1%). About one‐third of CHR‐P services (36.6%) implemented general public awareness campaigns, 63.4% implemented either a dedicated online site for service promotion or elaborated printed and other media materials (53.7%). Most CHR‐P services received young people from health‐related organizations, including outpatient or community mental health services (90.0%), and general healthcare services (75.0%). Education organizations were also frequent referral sources (65%) (Salazar de Pablo, Estradé, et al., 2021). Again, we think we can build on this evidence and think about new recruitment and characterization strategies, but we consider the knowledge achieved to be valuable.

Many individuals identified as CHR‐P exhibit persistent functional impairment and psychiatric comorbidities, such as mood and anxiety disorders, further underscoring the need for continued transdiagnostic clinical support beyond psychosis prediction. There has also been some focus on other outcomes that trigger help‐seeking behaviors, including negative symptoms and depressive symptoms, which are present and impairing in young people at CHR‐P, also in those who do not transition to psychosis after up to 16 years of follow‐up (Salazar de Pablo et al., 2022). The authors criticize the use or focus on negative symptoms, whereas at least 33 studies have been carried out on this category of symptoms on adolescents at CHR‐P (Salazar de Pablo et al., 2023). We believe the use of negative symptoms is not only correct but also beneficial for therapeutic purposes. In line with this, the dopamine hypothesis is not only a simplification of the experiences presented by young people with psychosis or at CHR‐P but also has limited clinical utility and relevance currently. Unsurprisingly, negative symptoms are defined as a reduction of normal functions related to either motivation and interest (e.g. avolition, anhedonia, and asociality) or expressive functions (e.g. blunted affect and alogia) (Correll & Schooler, 2020). These symptoms are present in most adolescents at CHR‐P (Salazar de Pablo et al., 2023) and are not simply the result of altered dopamine levels. In adolescents at CHR‐P, negative symptoms have been found to be clinically relevant and, in some cases, the predominant psychopathological feature. Negative symptoms are also associated with poor functioning and poor recovery levels in this group (Salazar de Pablo et al., 2023). Fortunately, our knowledge has advanced since the 1980s, to the point that some of the most promising pharmacological interventions for psychosis are not dopaminergic but have other mechanisms of action (e.g. glutamate, acetylcholine, neuroinflammation). In line with this commentary, we do not think the progress made thanks to the dopamine hypothesis should be abandoned, but we embrace the advancements in other areas. Remarkably, in the study by Miklowitz et al. referred to by Tiffin and Kelleher, negative symptoms were evaluated, and children and adolescents at CHR‐P who were on antipsychotics showed greater improvement in negative symptoms than those not on antipsychotics.

We were surprised by the selection of Miklowitz et al.'s (2014) study to criticize our approach since it was not included in our meta‐analytical analyses. For transparency, the figures in our manuscript report the effects of the interventions compared to the control groups on transition to psychosis rate, positive symptoms, negative symptoms, total symptoms, and functioning. It can be seen that Miklowitz et al.'s (2014) study was not included (Frearson et al., 2025). The reason for this is that it compares two active interventions, family‐focused therapy and psychoeducation (i.e., not one active intervention vs. no‐intervention as Tiffin and Kelleher seem to suggest). In the key findings, we do specify which of the active interventions was associated with differential changes in each subgroup, including the beneficial effect of psychoeducation on functioning for older adolescents compared to family‐focused therapy (Frearson et al., 2025). We agree psychoeducation may be sufficient to reduce the intensity and associated distress in adolescents at CHR‐P, and we have made this point before (Salazar de Pablo & Arango, 2023). In fact, for adolescents at CHR‐P and low risk, we have recommended psychoeducation strategies by specialist CAMHS or school mental health teams in a commentary in this journal (Salazar de Pablo & Arango, 2023). We have also repeatedly acknowledged the limited evidence favoring one specific treatment over the others to prevent psychosis, including psychoeducation and family‐focused interventions (Fusar‐Poli & Salazar de Pablo, 2022a; Salazar de Pablo & Arango, 2023; Salazar de Pablo, Estradé, et al., 2021).

In conclusion, we think we should refine and build on existing knowledge and evidence rather than simply abandoning the CHR‐P paradigm. Why discard a framework that has provided valuable insights when we have the opportunity to improve it? With advancements in genetics, neuroimaging, and machine learning, we can move beyond its current limitations, improving risk stratification and tailoring interventions to better serve individuals at risk. We can also expand the paradigm, so it supports other young people at risk, particularly those help‐seeking adolescents requiring input from CAMHS. While transition to psychosis remains a key outcome, broader mental health challenges in adolescents at CHR‐P (including negative symptoms) warrant continued support through transdiagnostic, developmentally sensitive interventions.

## Data Availability

Data sharing is not applicable to this article as no new data were created or analyzed.

## References

[camh12777-bib-0001] Correll, C.U. , & Schooler, N.R. (2020). Negative symptoms in schizophrenia: A review and clinical guide for recognition, assessment, and treatment. Neuropsychiatric Disease and Treatment, 16, 519–534. 32110026 10.2147/NDT.S225643 PMC7041437

[camh12777-bib-0002] Frearson, G. , de Otazu Olivares, J. , Catalan, A. , Aymerich, C. , & Salazar de Pablo, G. (2025). Review: Efficacy of preventative interventions for children and adolescents at clinical high risk of psychosis – A systematic review and meta‐analysis of intervention studies. Child and Adolescent Mental Health, 30, 66–82. 39688301 10.1111/camh.12755 PMC11754713

[camh12777-bib-0003] Fusar‐Poli, P. , & Salazar de Pablo, G. (2022a). Antipsychotics and attenuated psychosis syndrome: Transdiagnostic assessment and discontinuation strategies. Schizophrenia Research, 243, 402–404. 34217548 10.1016/j.schres.2021.06.030

[camh12777-bib-0004] Fusar‐Poli, P. , & Salazar de Pablo, G. (2022b). No meta‐analytic effect of age on probability of developing psychosis in individuals at clinical high risk. Journal of the American Academy of Child and Adolescent Psychiatry, 61, 731–732. 34929320 10.1016/j.jaac.2021.11.027

[camh12777-bib-0010] Miklowitz, D.J. , O'Brien, M.P. , Schlosser, D.A. , Addington, J. , Candan, K.A. , Marshall, C. , et al. (2014). Family‐focused treatment for adolescents and young adults at high risk for psychosis: results of a randomized trial. J Am Acad Child Adolesc Psychiatry, 53, 848‐858. 25062592 10.1016/j.jaac.2014.04.020 PMC4112074

[camh12777-bib-0005] Salazar de Pablo, G. , & Arango, C. (2023). Debate: Prevention of psychosis in adolescents – Does CAMHS have a role? Child and Adolescent Mental Health, 28, 550–552. 37424168 10.1111/camh.12662

[camh12777-bib-0006] Salazar de Pablo, G. , Catalan, A. , Vaquerizo Serrano, J. , Pedruzo, B. , Alameda, L. , Sandroni, V. , … & Correll, C.U. (2023). Negative symptoms in children and adolescents with early‐onset psychosis and at clinical high‐risk for psychosis: Systematic review and meta‐analysis. The British Journal of Psychiatry, 223, 282–294. 37194556 10.1192/bjp.2022.203 PMC10331322

[camh12777-bib-0007] Salazar de Pablo, G. , Estradé, A. , Cutroni, M. , Andlauer, O. , & Fusar‐Poli, P. (2021). Establishing a clinical service to prevent psychosis: What, how and when? Systematic review. Translational Psychiatry, 11, 43. 33441556 10.1038/s41398-020-01165-x PMC7807021

[camh12777-bib-0008] Salazar de Pablo, G. , Soardo, L. , Cabras, A. , Pereira, J. , Kaur, S. , Besana, F. , … & Fusar‐Poli, P. (2022). Clinical outcomes in individuals at clinical high risk of psychosis who do not transition to psychosis: A meta‐analysis. Epidemiology and Psychiatric Sciences, 31, e9. 35042573 10.1017/S2045796021000639 PMC8786617

[camh12777-bib-0009] Salazar de Pablo, G. , Woods, S.W. , Drymonitou, G. , de Diego, H. , & Fusar‐Poli, P. (2021). Prevalence of individuals at clinical high‐risk of psychosis in the general population and clinical samples. Systematic review and meta‐analysis. Brain Sciences, 11, 1544. 34827543 10.3390/brainsci11111544 PMC8615691

